# Nutrient data from US manure systems from 2012 to 2022: Spatial and temporal trends

**DOI:** 10.1002/jeq2.70268

**Published:** 2026-09-21

**Authors:** Nancy L. Bohl Bormann, Melissa L. Wilson, Erin L. Cortus, Kevin A. T. Silverstein, Kevin A. Janni, Larry M. Gunderson, Anna M. Cates, Paulo H. Pagliari

**Affiliations:** ^1^ Soil, Water, & Climate Department University of Minnesota‐Twin Cities Saint Paul Minnesota USA; ^2^ Bioproducts & Biosystems Engineering Department University of Minnesota‐Twin Cities Saint Paul Minnesota USA; ^3^ Research Computing University of Minnesota‐Twin Cities Minneapolis Minnesota USA; ^4^ Minnesota Department of Agriculture Pesticide & Fertilizer Management Saint Paul Minnesota USA

## Abstract

Most published manure nutrient concentration approximations (book values) used today were derived from manure data prior to 2003 and do not reflect potential differences across US regions. Using ManureDB data, the University of Minnesota database with manure total nitrogen (N), ammonium‐N (NH_4_‐N), phosphorus (P_2_O_5_), and potassium (K_2_O) data spanning from 1998 to 2023 from 15 laboratories across the United States, we analyzed nutrient data from 2012 to 2022. Eight common US poultry and livestock manure types were used in this analysis, which showed that 91% of the slurry/liquid and 66% of the semisolid/solid manure nutrient categories had significant regional differences. Liquid swine and liquid dairy manure in the Southeast (SE) region had significantly lower nutrient data than the Northeast (NE) and US Midwest (MW) regions every year for swine and 10 out of 11 years for dairy. Nutrient concentrations in the solid chicken manures were the most temporally volatile across the decade with 38% of the region–analyte combinations having significant trends. This was followed by liquid dairy (33%), solid dairy (25%), solid turkey (25%), solid beef (8%), liquid swine (8.3%), and liquid beef (no trends). The frequency of significant manure nutrient trends in the NE and SE regions was triple that of the MW region. Decreasing manure nutrient concentrations were four times more prevalent than increasing nutrient concentrations across 2012–2022, perhaps displaying the impact of precision animal nutrition on manure nutrient content. Updated US manure nutrient characteristics provide a revised baseline for farmers, consultants, and researchers, while simultaneously highlighting manure nutrient variability.

## Plain Language Summary

Most published manure nutrient approximations used today came from manure samples prior to 2003 and do not reflect possible differences across US regions. Using ManureDB data, the University of Minnesota manure nutrient database, we analyzed nutrient data from 2012 to 2022. Eight common US poultry and livestock manure types were used in this analysis, which showed that 91% of the liquid and 66% of the solid animal manure nutrient categories had significant regional differences. Solid manures showed significant nutrient trends 25% of the time, but only 18% for liquid manures. The Northeast and Southeast regions had three times more significant trends than the US Midwest. Decreasing manure nutrient concentrations were four times more common than increasing nutrient concentrations. Having current values of US manure nutrients gives an improved baseline of values for farmers, consultants, and researchers, while highlighting the variability in manure nutrients.

**Abbreviations**
ASABE: American Society of Agricultural and Biological Engineers
IQR: interquartile range
MAD: median absolute deviation
MW: Midwest
MWPS: Midwest Plan Service
NE: Northeast
RMD: relative median deviation
SE: Southeast

## 1 INTRODUCTION

Manure provides nutrients for crops when land applied; however, if manure N or P is applied beyond what the crop can utilize or not incorporated into the soil, these nutrients can be lost to the environment. Manure nutrients can be inconsistent due to animal species, age, diet, management, housing, storage, treatment, and climate. Manure analyses provide information to calculate manure application rates to meet crop nutrient needs and minimize nutrient accumulation or losses to the environment. Book values are an alternative when actual manure samples are not available, such as for agricultural and environmental modeling efforts, planning new animal feeding operations or existing operation changes, or regulatory targets, usually focusing on the major nutrients of total N, P_2_O_5_, K_2_O, and the total N component NH_4_‐N. Several commonly cited manure nutrient book values include Midwest Plan Service (MWPS) from 2004, American Society of Agricultural and Biological Engineers (ASABE) from 2005, and United States Department of Agriculture—Natural Resources Conservation Service from 2008 (ASABE, 2005; Lorimor et al., 2004; USDA‐NRCS, 2008). These resources of as‐stored manure nutrient approximations are two decades old and may not reflect current agricultural production practices or operations from across the country.

A more comprehensive and modern source of manure nutrient data was recently released: ManureDB, a national database of manure nutrients from agricultural laboratories across the United States (Bohl Bormann et al., 2024). The database was published in 2023 with data attributed to specific regions across the United States and spanning back to 1998. This allowed for a unique opportunity to evaluate manure nutrient characteristics for temporal and spatial changes on a national scale. The objectives of this paper were to (1) evaluate regional differences (for regions with sufficient samples) and (2) determine if there were temporal trends for four analytes (total N, NH_4_‐N, P_2_O_5_, and K_2_O). Our goal was to provide a broad overview and current characterizations of manure nutrients. It should be noted that the database does not include or predict manure production volumes.

## 2 METHODS

### 2.1 ManureDB data

As of February 2024, ManureDB aggregated 5.2 million data points from 489,034 samples spanning 55 animal or other amendment types and 15 laboratories. The background and design of ManureDB can be found in Bohl Bormann et al. (2025) including more specific details on data input and cleaning. Relevant details are summarized here:

1. A database display function rule required at least five samples of an animal type per year from a location (state, region, or nation) to be included in the dataset (i.e., data not meeting this requirement were not included in the dataset).
2. Analyte units and basis (as‐received [wet] or dry) were standardized in the summary using equations from the *Recommended methods of manure analysis, second edition* (Wilson et al., 2022).
3. For each analyte, different analytical methods may have been used and were treated as equivalent (i.e., total Kjeldahl N and combustion methods for determining total N were considered equivalent, so both were reported as “total N”).
4. To categorize samples by moisture content, moisture designations were applied as determined by the MWPS definitions (Lorimor et al., 2004) or based on sample analysis as described in Bohl Bormann et al. (2025).
5. Animal or other amendment type was determined from common livestock and poultry species, and then more specific life stages or genders were separated out if it could be determined from the laboratory submittal sheets.

### 2.2 Data selection

In this paper we focused on the highest value livestock and poultry commodities (USDA‐APHIS, 2016). The eight animal manure categories selected were solid beef, solid chicken broiler, solid chicken layer, solid dairy, solid turkey, liquid beef, liquid dairy, and liquid swine manure. Because of varying degrees of sample descriptions across all the sources, no differentiation was shown in this paper between manure storage systems, life stage, bedding, agitation, treatment, or storage length. While the database contained data from 1998 to 2023, the period from 2012 to 2022 was selected for its relevance to current conditions and density of data. Since manure, particularly storage and handling, is affected by climate, we grouped the data according to USDA Climate Hub regions (USDA, 2020): US Midwest (MW), Northeast (NE), Northern Plains, Pacific Northwest, Southeast (SE), Southern Plains, and Southwest (Table 1).

**TABLE 1:** States in the seven USDA Climate Hub regions.

Region	State
US Midwest (MW)	IA, IN, IL, MI, MN, MO, OH, WI
Northeast (NE)	CT, DE, MA, MD, ME, NH, NJ, NY, PA, RI, VT, WV
Northern Plains (NP)	CO, MT, ND, NE, SD, WY
Pacific Northwest (PN)	AK, ID, OR, WA
Southeast (SE)	AL, AR, DC, GA, FL, KY, LA, MS, NC, PR, SC, TN, VA
Southern Plains (SP)	KS, OK, TX
Southwest (SW)	AZ, CA, HI, NM, NV, UT

Total N, NH_4_‐N, P_2_O_5_, and K_2_O were selected for data analysis, as they are the primary nutrients of concern for crop producers utilizing manure. Manure nutrient units were on an as‐received (wet) basis, as that reflects how manure is handled and how application rates are calculated in the United States. Median, median absolute deviation (MAD), relative median deviation (RMD), 25th percentile, 75th percentile, and count were calculated for total N, NH_4_‐N, P_2_O_5_, and K_2_O for each of the eight animal manure categories by region and category. MAD was used to measure dataset variability and is less influenced by outliers compared to standard deviation. MAD is calculated by finding the median of a dataset, subtracting the median from each value in the dataset, and then finding the median from those calculations. Like a coefficient of variation, the RMD is a dimensionless number that indicates method precision and is calculated by dividing the MAD by the median and multiplying by 100. The 25th and 75th percentiles are the first and third quartiles, respectively. The base R package was utilized for these analyses (R Core Team, 2023).

**Core ideas**

- US regional differences were detected for most common animal manure types.
- From 2012 to 2022, solid manures showed significant temporal trends 25% of the time, but only 18% for liquid manures.
- ManureDB offered time and region‐specific manure nutrient data for decision‐making.

### 2.3 Analysis by region and manure type

We compared medians between regions for animal manure categories for each year. Eight animal manure categories and four analytes resulted in 32 separate datasets. To ensure sufficient statistical power for trend detection, regional comparisons were limited to datasets with a cumulative minimum of 500 samples with annual representation across the 2012–2022 period. Regions with over 500 samples but not samples each year are also included in the summary tables for each animal manure category in the Supporting Information (Tables S1–S5). We observed positive right‐skewed data in 31 of 32 datasets. The ManureDB means were larger than the medians 85% of the time, indicating some larger outliers elevated the means. Multiple data transformations were attempted, and the data could not be normalized through those transformations. The Jarque–Bera test for normality confirmed the datasets (each analyte for a specific animal manure category) were not normally distributed by using jarque.bera.test function in the “tseries” package (Jarque & Bera, 1987; Trapletti et al., 2023). The wilcox_test function was used to complete the nonparametric Mann–Whitney *U* test along with the p.adjust function applied to the *p*‐value for the Benjamini–Hochberg (false discovery rate) correction with both functions from the R package “coin” (Benjamini & Hochberg, 1995; Hothorn et al., 2023; Mann & Whitney, 1947).

### 2.4 Analyte trends over time

Separate animal manure category datasets for each region with at least 500 samples including each year from 2012 to 2022 were analyzed for trends across that time span. The SE, MW, and NE regions had seven, six, and five animal manure categories that met those parameters, respectively. The nonparametric Mann–Kendall trend test was used for those regions with sufficient samples for each of the eight animal manure categories using the “MannKendall” function in R (Kendall, 1955; Mann, 1945; McLeod, 2022). A test statistic and two‐sided *p*‐values were calculated for each animal manure category, region, and analyte combination indicating no significant trend, a significant increasing trend, or a significant decreasing trend. Line graphs for medians and interquartile ranges (IQRs) of each analyte per year and region for each animal manure category with sufficient samples were made utilizing the “ggplot2” package (Wickham et al., 2024).

## 3 RESULTS AND DISCUSSION

### 3.1 Regional comparisons

**FIGURE 1:**
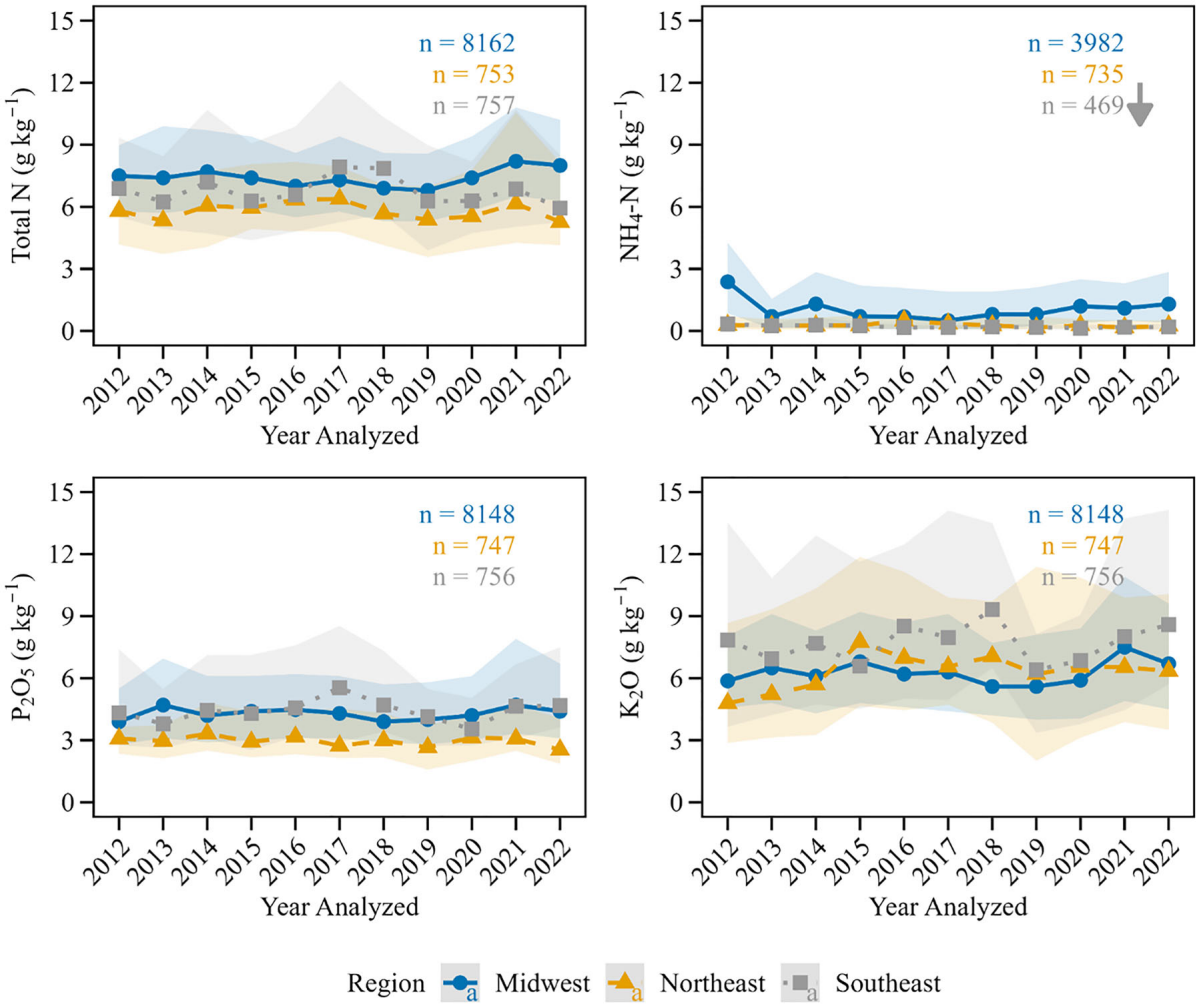
Solid beef manure total N, NH_4_‐N, P_2_O_5_, and K_2_O medians from 2012 to 2022 for the US Midwest, Northeast, and Southeast regions. Only regions with at least 500 samples and samples for each year were included in this analysis. *n* = sample count by region. The shaded areas show the interquartile range (IQR) by regions differentiated by color. **↓** indicates a significant decreasing trend.

**FIGURE 2:**
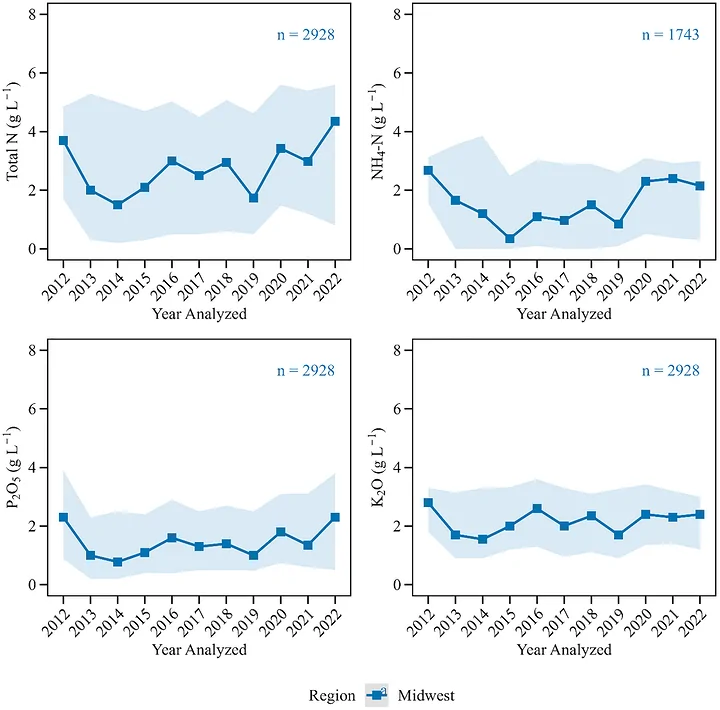
Liquid beef manure total N, NH_4_‐N, P_2_O_5_, and K_2_O medians from 2012 to 2022 for the US Midwest region. Only regions with at least 500 samples and samples for each year were included in this analysis. *n* = sample count by region. The shaded areas show the interquartile range (IQR) by region. No significant analyte trends.

**FIGURE 3:**
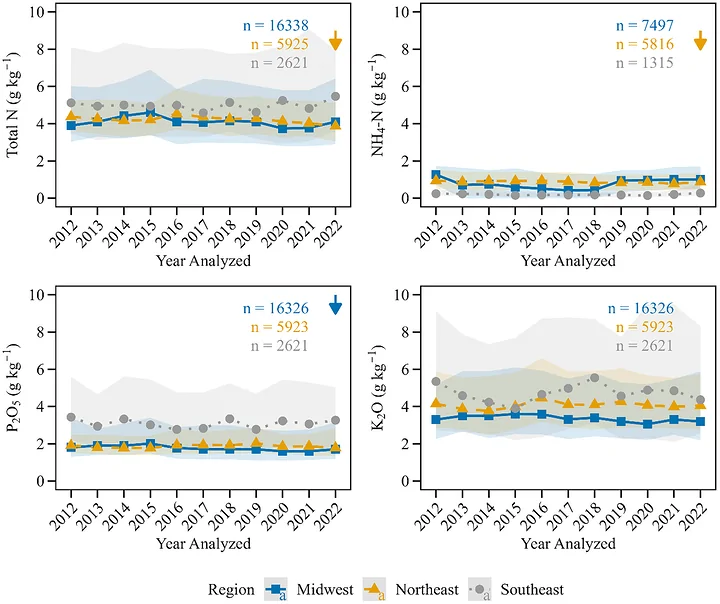
Solid dairy manure total N, NH_4_‐N, P_2_O_5_, and K_2_O medians from 2012 to 2022 for the US Midwest, Northeast, and Southeast regions. Only regions with at least 500 samples and samples for each year were included in this analysis. *n* = sample count by region. The shaded areas show the interquartile range (IQR) by regions differentiated by color. **↓** indicates a significant decreasing trend.

**FIGURE 4:**
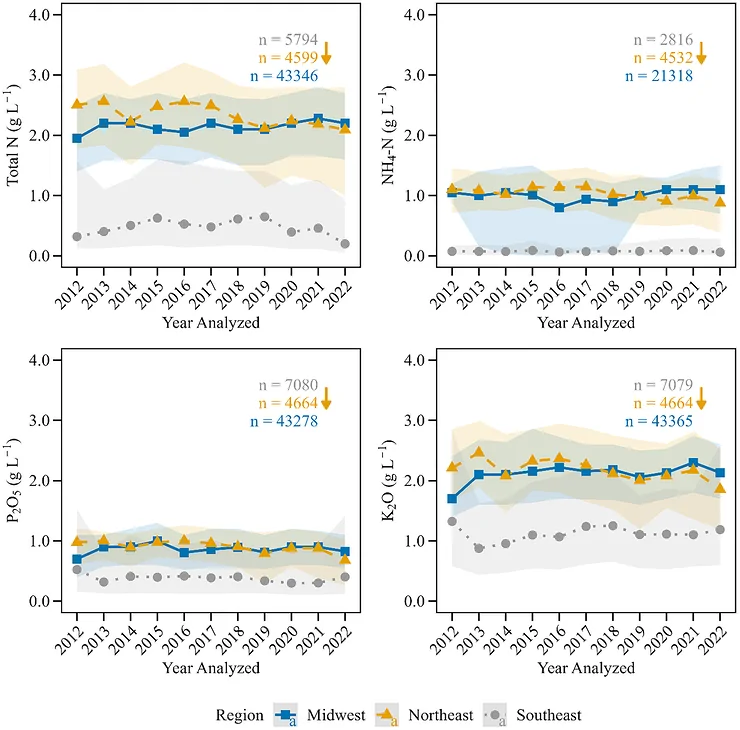
Liquid dairy liquid manure total N, NH_4_‐N, P_2_O_5_, and K_2_O medians from 2012 to 2022 for the US Midwest, Northeast, and Southeast regions. Only regions with at least 500 samples and samples for each year were included in this analysis. *n* = sample count by region. The shaded areas show the interquartile range (IQR) by regions differentiated by color. **↓** indicates a significant decreasing trend.

**FIGURE 5:**
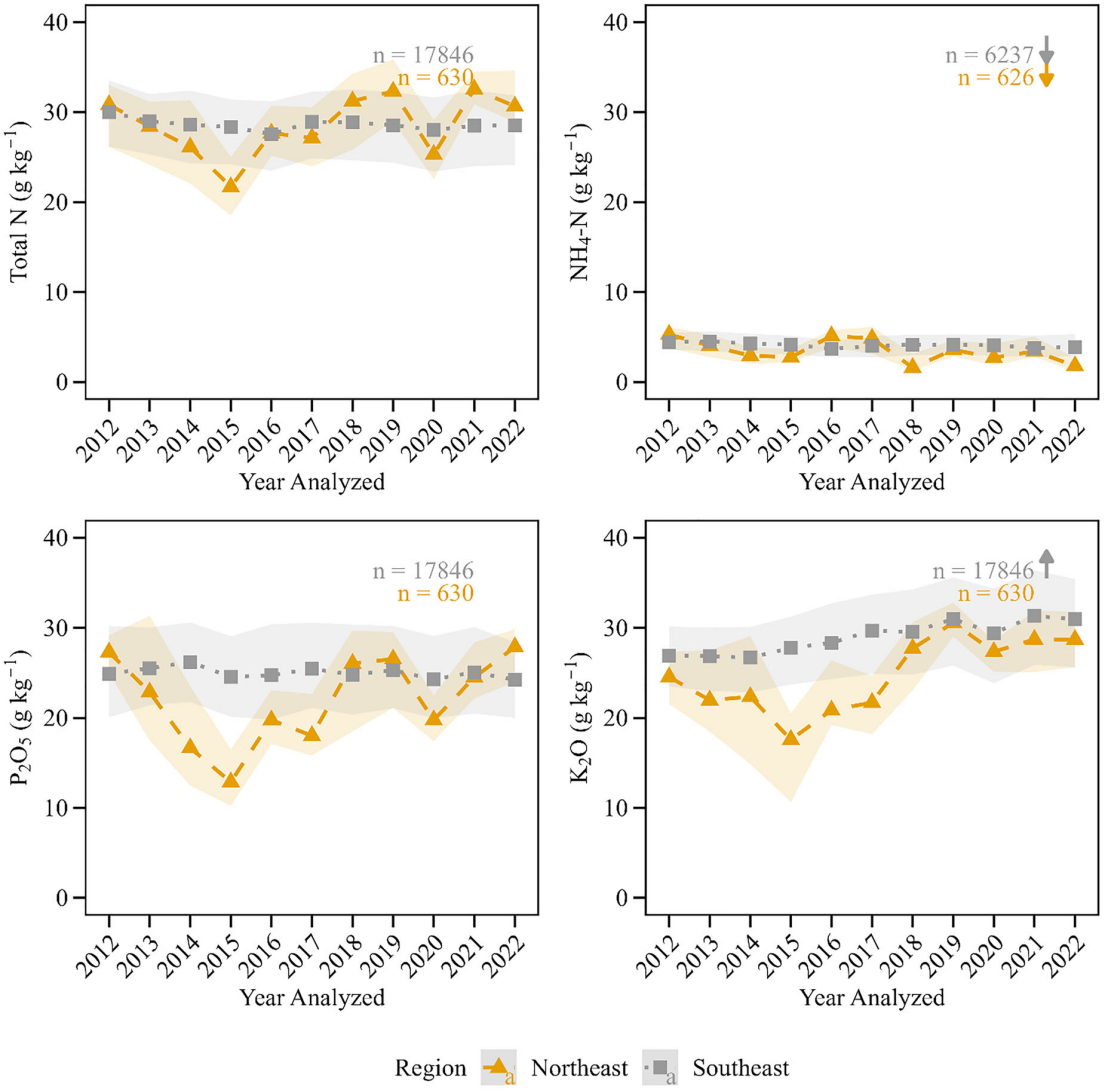
Solid chicken broiler manure total N, NH_4_‐N, P_2_O_5_, and K_2_O medians from 2012 to 2022 for the Northeast and Southeast regions. Only regions with at least 500 samples and samples for each year were included in this analysis. *n* = sample count by region. The shaded areas show the interquartile range (IQR) by regions differentiated by color. **↓** indicates a significant decreasing trend and ↑ indicates a significant increasing trend.

**FIGURE 6:**
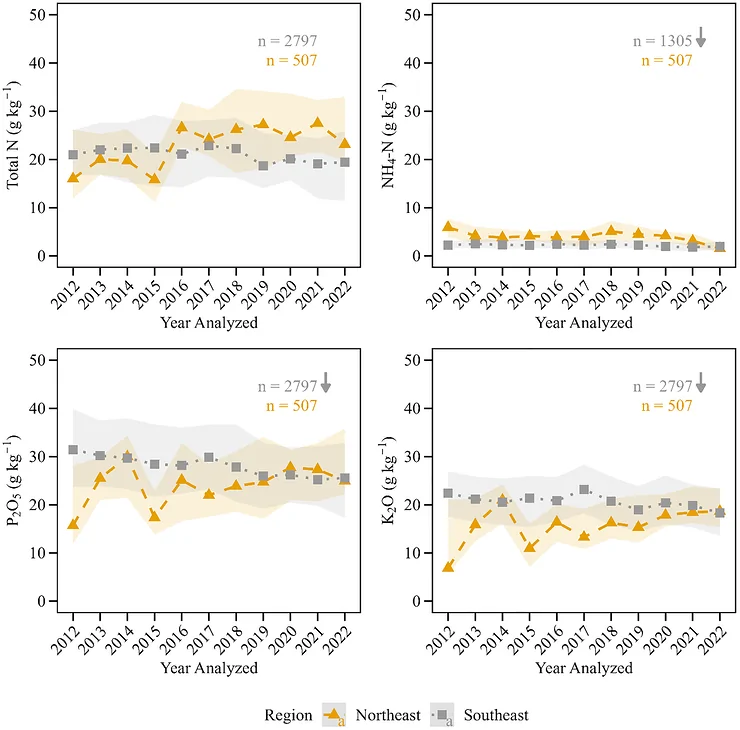
Solid chicken layer solid manure total N, NH_4_‐N, P_2_O_5_, and K_2_O medians from 2012 to 2022 for the Northeast and Southeast regions. Only regions with at least 500 samples and samples for each year were included in this analysis. *n* = sample count by region. The shaded areas show the interquartile range (IQR) by regions differentiated by color. **↓** indicates a significant decreasing trend.

**FIGURE 7:**
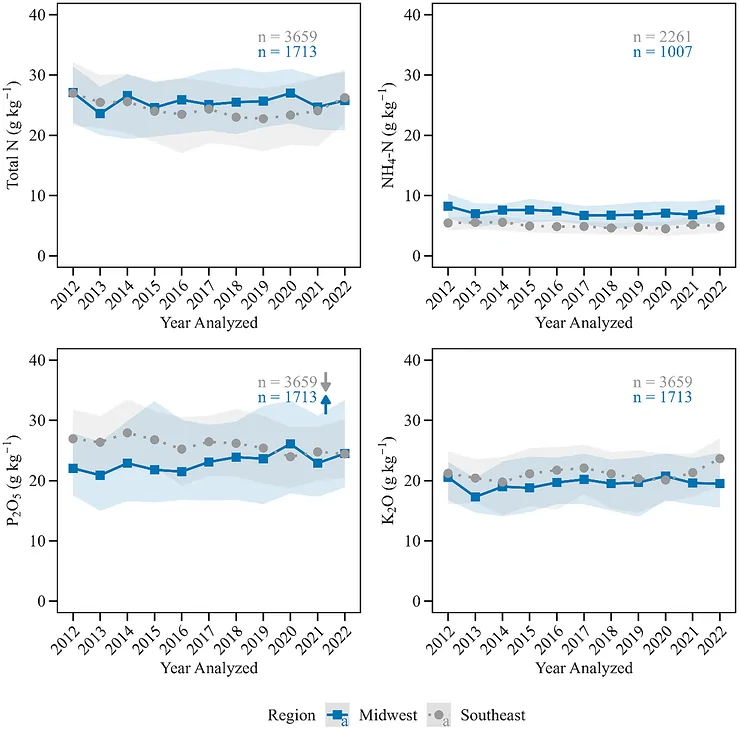
Solid turkey manure total N, NH_4_‐N, P_2_O_5_, and K_2_O medians from 2012 to 2022 for the US Midwest and Southeast regions. Only regions with at least 500 samples and samples for each year were included in this analysis. *n* = sample count by region. The shaded areas show the interquartile range (IQR) by regions differentiated by color. **↓** indicates a significant decreasing trend and **↑** indicates a significant increasing trend.

**FIGURE 8:**
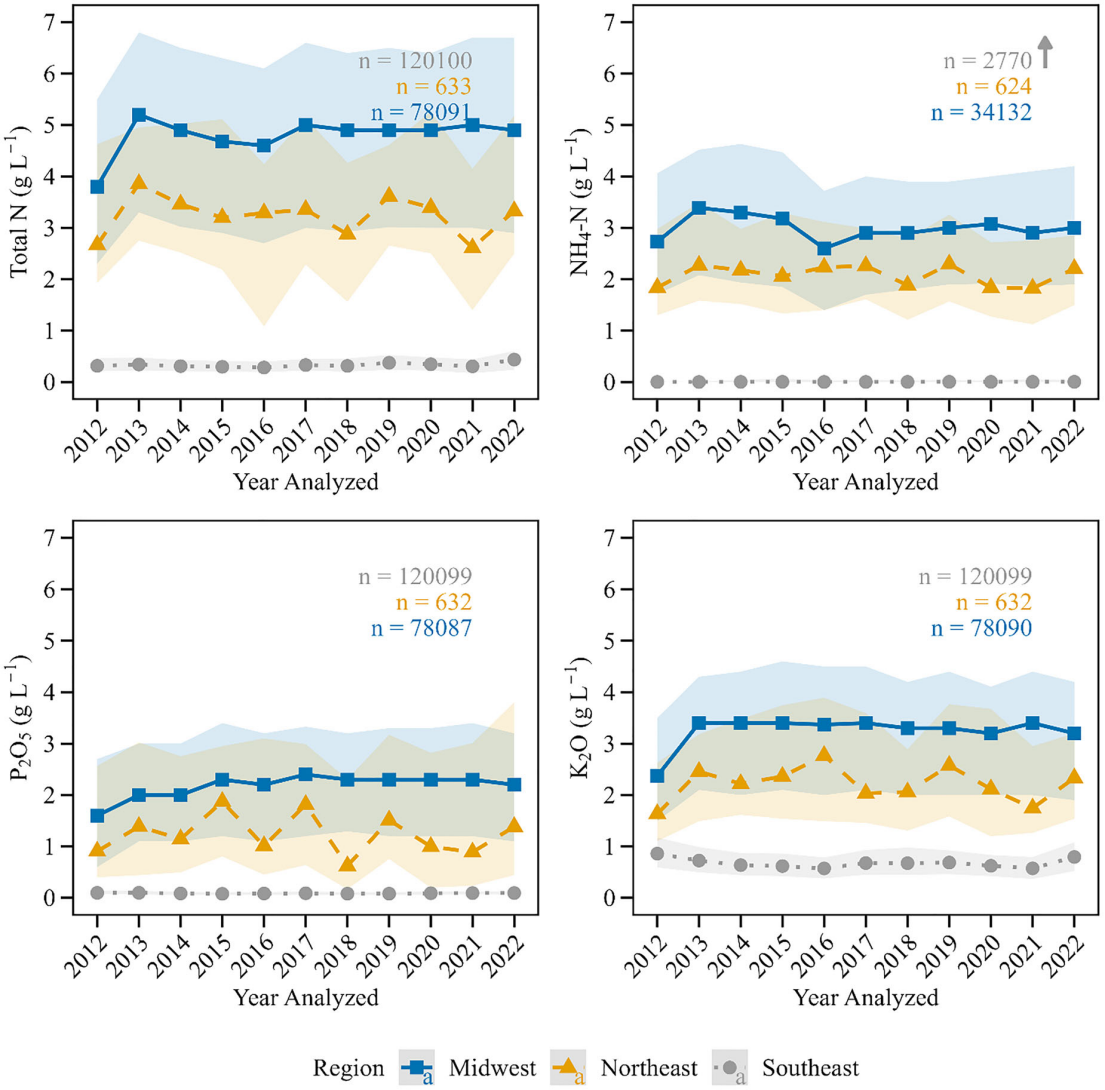
Liquid swine manure total N, NH_4_‐N, P_2_O_5_, and K_2_O medians from 2012 to 2022 for the US Midwest, Northeast, and Southeast regions. Only regions with at least 500 samples and samples for each year were included in this analysis. *n* = sample count by region. The shaded areas show the interquartile range (IQR) by regions differentiated by color. **↑** indicates a significant increasing trend.

Manure medians and IQRs across 2012–2022 for total N, NH_4_‐N, P_2_O_5_, and K_2_O for each of the eight animal manure categories by region are shown in Figures 1, 2, 3, 4, 5, 6, 7, 8 and Tables S1–S5. Figures 1, 2, 3, 4, 5, 6, 7, 8 also include arrows to indicate significant (*p* < 0.05) increasing or decreasing nutrient trends. Most solid manure nutrients tended to have regional differences. Regional comparisons of solid manure analytes for individual years (e.g., 2012 MW to 2012 SE) revealed that 66% (260 of 296) were significantly different (Table 2). For 36 comparisons over the 11‐year period shown in Table 2, only three comparisons for two nutrients were not significantly different (MW‐SE for solid beef P_2_O_5,_ MW‐NE for solid beef K_2_O, and NE‐SE for solid dairy K_2_O). Most liquid manure nutrients also tended to have regional differences. For individual year comparisons for liquid manure analytes between regions, 240 out of 264 comparisons (91%) were significantly (*p* < 0.05) different (Table 3). In fact, of 24 comparisons over the 11‐year period shown in Table 3, only two comparisons for two nutrients were not significantly different (MW‐NE for liquid dairy P_2_O_5_ and K_2_O). This analysis demonstrated that regional liquid manure nutrient concentrations are a closer nutrient approximation than a US median or average.

**TABLE 2:** Regional total N, NH_4_‐N, P_2_O_5_, and K_2_O comparisons for solid beef, chicken broiler, chicken layer, dairy, and turkey manure categories 2012–2022^a^. Only regions with at least 500 samples with annual representation were included in this analysis.

Manure type	Analyte	Regional comparison	% of years significantly different
Solid beef	Total N	MW‐NE	91
		NE‐SE	64
		MW‐SE	45
	NH_4_‐N	MW‐NE	91
		NE‐SE	27
		MW‐SE	100
	P_2_O_5_	MW‐NE	100
		NE‐SE	91
		MW‐SE	9
	K_2_O	MW‐NE	9
		NE‐SE	36
		MW‐SE	45
Solid chicken broiler	Total N	NE‐SE	55
	NH_4_‐N		64
	P_2_O_5_		55
	K_2_O		82
Solid chicken layer	Total N	NE‐SE	73
	NH_4_‐N		91
	P_2_O_5_		36
	K_2_O		45
Solid dairy	Total N	MW‐NE	64
		NE‐SE	91
		MW‐SE	91
	NH_4_‐N	MW‐NE	82
		NE‐SE	100
		MW‐SE	100
	P_2_O_5_	MW‐NE	73
		NE‐SE	100
		MW‐SE	100
	K_2_O	MW‐NE	73
		NE‐SE	18
		MW‐SE	45
Solid turkey	Total N	MW‐SE	27
	NH_4_‐N	MW‐SE	100
	P_2_O_5_	MW‐SE	55
	K_2_O	MW‐SE	27

Abbreviations: MW, US Midwest; NE, Northeast; SE, Southeast.

^a^ ManureDB (Bohl Bormann et al., 2024b).

**TABLE 3:** Regional total N, NH_4_‐N, P_2_O_5_, and K_2_O comparisons for liquid dairy and swine manure categories 2012–2022^a^. Only regions with at least 500 samples with annual representation were included in this analysis.

Manure	Analyte	Regional comparison	% of years significantly different
Liquid dairy	Total N	MW‐NE	64
		NE‐SE	100
		MW‐SE	100
	NH_4_‐N	MW‐NE	73
		NE‐SE	100
		MW‐SE	100
	P_2_O_5_	MW‐NE	55
		NE‐SE	91
		MW‐SE	100
	K_2_O	MW‐NE	64
		NE‐SE	100
		MW‐SE	100
Liquid swine	Total N	MW‐NE	100
		NE‐SE	100
		MW‐SE	100
	NH_4_‐N	MW‐NE	91
		NE‐SE	100
		MW‐SE	100
	P_2_O_5_	MW‐NE	55
		NE‐SE	100
		MW‐SE	100
	K_2_O	MW‐NE	100
		NE‐SE	100
		MW‐SE	100

Abbreviations: MW, US Midwest; NE, Northeast; SE, Southeast.

^a^ ManureDB (Bohl Bormann et al., 2024b).

#### 3.1.1 Solid beef manure

The MW, NE, and SE regions each had at least 500 solid beef manure samples with annual representation from 2012 to 2022. The NE region consistently exhibited lower medians for total N and P_2_O_5_ across all years. Across years, the NE total N median was 23% lower than the MW and 15% lower than SE. The MW region had significantly higher (*p* < 0.05) total N medians than the NE region in 91% of years and significantly higher total N medians than the SE region in 5 of 11 years (Table 2). For NH_4_‐N, the MW consistently had significant differences with 400% higher medians than the SE every year. Compared to the NE, the MW NH_4_‐N median was significantly greater in 10 of 11 years, with 257% higher medians. For P_2_O_5_, the MW region had significantly greater medians than the NE in all years (43% higher), while the SE region had significantly greater medians than the NE 91% of the time with medians 47% higher. The SE region had significantly greater P_2_O_5_ than the MW region in only 1 of 11 years. For K_2_O, MW and NE regional comparisons were not significantly different, except for 1 year. The SE region had significantly greater K_2_O than the NE region 36% of the years with a median 19% greater than the NE. The SE region was greater than the MW region 45% of years with a 19% larger median. The relative consistency in potassium levels across regions could be because potassium is not as mobile or volatile as the other analytes.

Regional differences in solid beef manure could stem from bedding type and inclusion rate, climate, manure storage and handling, and diet. With the MW leading in corn (*Zea mays*) and soybean (*Glycine max*) production, those grains are commonly incorporated into cattle diets, along with dried distillers grains as a byproduct of corn ethanol production (Benke et al., 2010; Regassa et al., 2008). Southern US cotton (*Gossypium hirsutum*) and peanut (*Arachis hypogaea*) growing states may have by‐products of those crops available for beef feedstuffs (Myer & Hersom, 2021; Stewart & Rossi, 2022). The southern states have longer growing seasons for forages available, whereas the northern states may rely more on hay or other dried grain and roughage. Forages contain a higher potassium content than grain, and in this dataset the SE region had higher manure K_2_O levels than the other two regions. On the other hand, when considering climate, the warmer temperatures in the southern United States increase ammonia volatilization from manure, which was observed by the lower NH_4_‐N manure content in the SE region compared to the NE and MW regions.

#### 3.1.2 Liquid beef manure

For the liquid beef manure category, the MW region was the only region to have samples each year across 2012–2022 and at least 500 samples. This suggested that deep pit beef farms are more common in the MW relative to other regions. Regardless, farms may sample their solid beef manure more frequently than sampling their runoff holding area due to the larger manure quantities and nutrient concentrations of solid manure. Some beef operations have head capacities under the regulated farm size (Gollehon et al., 2001), and therefore they are less likely to have nutrient management plans requiring or encouraging manure testing as well.

#### 3.1.3 Solid dairy manure

Three regions (MW, NE, and SE) had sufficient solid dairy manure samples across 2012–2022. The solid dairy samples had the greatest differences between years and regions (79%) compared to other solid manure types. The SE region had greater total N, P_2_O_5_, and K_2_O and lower NH_4_‐N than the MW or NE regions. Across 10 of the 11 years analyzed, the SE region exhibited significantly greater total N median concentrations with 22% greater than the MW and 19% greater than in the NE. The SE also had significantly lower NH_4_‐N and greater P_2_O_5_ for all years compared to the MW (−77%, +55%) and NE (−80%, +47%), respectively. Lower ammonium and higher P might indicate more decomposition due to a warmer climate and/or more composting. The closest analyte between regions was within the NE‐SE K_2_O comparisons, where the SE region was significantly greater in only 2 out of 11 years. Dairy cattle manure can be segmented by life stage and/or sex from dairy calves, heifers, dairy steers, to lactating or dry cows. The dairy cattle could be housed indoors with various amounts and types of bedding mixed with manure or outdoors where manure is scraped and piled, or a combination of both. Like beef production, dairies can vary in the grain and forage proportions of their diets regionally. Dairy manure could also go through a manure separation, digestion, and/or composting processes resulting in altered manure components. All these possibilities contribute to the high nutrient variability in solid dairy manure.

#### 3.1.4 Liquid dairy manure

Six of the seven US regions had over 500 samples for liquid dairy manure; however, only the MW, NE, and SE regions met the data requirements for analysis. All analytes in the MW region were significantly greater than the SE region every year as shown in Table 3 and Table S2. The MW region had a 368% larger total N, 1247% larger NH_4_‐N, 134% larger P_2_O_5_, and 89% larger K_2_O medians than the SE. All the analytes in the NE region were also significantly greater than the SE in every instance aside from 1 year of P_2_O_5_ with medians 389%, 1302%, 140%, and 98% greater for total N, NH_4_‐N, P_2_O_5,_ and K_2_O, respectively. Anaerobic lagoons are more prevalent in the Southeastern United States due to a climate where they can function year‐round (Waldrip et al., 2020). Since those lagoons reduce nitrogen through denitrification, they can contribute to the large differences in total N and NH_4_‐N concentrations in the SE compared to the NE or MW. When all the years were combined, the MW‐NE regions were not significantly different for P_2_O_5_ or K_2_O. This may be due to similar manure storage structures and climates. However, the total N and NH_4_‐N values were significantly different between the NE‐MW regions 64% and 73% of the years, respectively, with the NE having 5% larger total N and 4% greater NH_4_‐N concentrations.

Animal health affects manure quantity and composition as stressed animals can have varying feed intake and altered digestion patterns (Kyriazakis et al., 1998). Heat stress for the dairy cattle is a larger concern in the warmer southern states. Feeding management strategies can differ between operations with some dairies using management‐intensive feeding systems, component feeding systems, or total mixed ration feeding systems (Njuki, 2022). Different animal breeds can have significantly different manure nutrient composition as Knowlton et al. (2010) demonstrated in their study of Holstein and Jersey dairy cows. More intensively managed operations may also be using additional manure treatment and handling technologies, which could impact the nutrient distribution in the manure. For example, the concentrations of Total N, NH_4_‐N, HPO_4_, K, Ca, and other nutrients in manure, tend to be higher in the liquid phase compared with the solid phase after undergoing liquid–solid separation (Pagliari & Laboski, 2013). The same authors also reported that the opposite could also occur where depending on a nutrient's solubility, nutrients could precipitate out of solution and remain in the solid phase.

#### 3.1.5 Solid chicken broiler manure

The NE and SE regions had enough samples for broiler comparisons and were 64% significantly different across 2012–2022. The SE region usually had greater analyte values, with 4%, 21%, 19%, and 17% greater medians for total N, NH_4_‐N, P_2_O_5_, and K_2_O, respectively, than the NE. The SE region had more samples (17,846) and less median fluctuation compared to the NE region (630). The SE region leads broiler production in the United States and also has larger broiler confinements compared to other regions (Perry et al., 1999). Several integrators dominate the broiler industry and influence the management (e.g., feed and veterinary care) and facilities used for broiler production (Bryant et al., 2022; MacDonald, 2008). With 95% of broilers contractually produced, similar management would be expected in this industry compared to other livestock and poultry industries (National Chicken Council, 2024).

#### 3.1.6 Solid chicken layer manure

The NE and SE regions had sufficient solid layer manure samples for comparison. Total N was significantly different between the regions 73% of the time (Table 2). The NE region had greater total N levels after 2015, but it is unclear why that occurred (Figure 6). Over the 11‐year time frame, the SE had −9% lower total N median than the NE. For NH_4_‐N, however, the SE region was significantly lower than the NE 10 out of 11 years with a median −47% lower than the NE overall for 2012–2022. This was likely due to warmer temperatures encouraging greater volatilization in the SE. Manure P_2_O_5_ was more similar across regions, with only 36% of the years significantly different and the SE having a 19% greater median than the NE. The NE region had lower K_2_O every year with 5 out of 11 years significantly lower. The SE region had 16% higher K_2_O over the 11 years. Climate, bedding usage and type, feed ingredients, manure treatments, housing, and manure management can all impact layer manure.

#### 3.1.7 Solid turkey manure

The MW and SE regions had sufficient turkey samples to compare and only 3 out of 11 years were significantly different for total N (Table 2). The SE 11‐year medians were −4% total N, −30% NH_4_‐N, +13% P_2_O_5_, and +5% K_2_O different than the MW medians. MW NH_4_‐N levels were significantly greater than SE region in all years, again likely due to the warmer temperatures in the SE region encouraging ammonia volatilization. Six out of 11 years the SE region had significantly greater P_2_O_5_ medians than the MW region. Eight of the 11 years the regions were not significantly different for K_2_O medians. The solid turkey samples had the least differences between years and regions (52%) of all the animal manure types. Turkeys generally have more similar housing and manure management compared to the animal types (Waldrip et al., 2020). Turkeys are usually raised in enclosed barns with organic bedding (e.g., wood shavings, rice (*Oryza sativa*) hulls, or chopped straw) on the floor. Turkey litter treatment adoption, type of treatments utilized, bedding materials, and feed formulations may vary regionally.

#### 3.1.8 Liquid swine manure

The MW, NE, and SE regions had enough samples for liquid swine comparisons. All analyte and year comparisons were significantly different except 1 year for NH_4_‐N and 5 years for P_2_O_5_ between the MW and NE regions (Table 3). The NE medians were lower across all analytes than the MW for all years: −33% total N, −30% NH_4_‐N, −45% P_2_O_5_, and −36% K_2_O. Due to the prevalence of anaerobic treatment lagoons in the SE, as compared to deep pits in the NE and MW, the SE region had lower median values for all four analytes: total N, NH_4_‐N, P_2_O_5,_ and K_2_O (−93, −100%, −96%, −80% for the MW and −90%, −100%, −93%, −68% for the NE, respectively) (Key et al., 2011). The SE region dominated ManureDB's liquid swine manure category with 55% of the samples. Since the swine category dominates ManureDB with over 199,000 samples, there is room for future dissections of these values into more detailed categories.

### 3.2 Regional trends over time

The 2012–2022 trends for analyte–animal manure categories for each region are shown within Figures 1, 2, 3, 4, 5, 6, 7, 8. The MW region had significant trends for 8% of the analyte–animal manure categories, the NE had significant trends for 29% of the analyte–manure categories, and the SE had significant trends for 28% of the analyte–manure categories. Among solid samples there were significant analyte trends 25% of the time. The liquid samples had significant trends 18% of the time.

The NH_4_‐N in solid beef, solid broiler, and solid layer manure all declined while the liquid swine manure NH_4_‐N increased in the SE region. The P_2_O_5_ declined in both SE solid layer and turkey manure. The K_2_O declined for SE solid layer and increased for SE solid broiler manure. Solid dairy manure total N and NH_4_‐N and solid broiler NH_4_‐N all declined in the NE. All four analytes in liquid dairy manure declined in the NE region. The P_2_O_5_ declined in MW solid dairy manure. For MW turkeys, P_2_O_5_ increased. No trend was observed in any analyte for the liquid beef samples. Animal nutrition continues to improve in efficiency, which could contribute to decreasing manure nutrient concentrations. The closer the nutrients match the animal's dietary needs, the less nutrients in the resulting manure. Ingredient availability and cost may result in overfeeding certain nutrients, which translate into excess nutrients excreted in the manure. The SE region had the only liquid swine manure trend with an increase for NH_4_‐N. However, those SE region NH_4_‐N values were extremely small with the median of 3.1 mg L^−1^ NH_4_‐N. More investigation is needed to determine if the trends were resulting from changing diets, treatments, animal housing and manure storages, or climatic trends or a combination. Annual additions to ManureDB could assess if these trends continue or level off.

## 4 CONCLUSIONS

Livestock and poultry production practices vary across the United States with climate, land, feed, labor availability, management, regulatory requirements, and market options. Regional differences were observed most of the years for every animal manure category, especially for the liquid manures. Differing climates and manure storage systems seem to drive these regional differences, especially in the SE region. Significant analyte trends were observed 22% of the time for all the region–animal manure categories spanning 2012–2022, with the majority decreasing. Continued improvement in animal nutrition reduces overfeeding of nutrients resulting in lower manure nutrient concentrations. With over 325,000 samples analyzed for the animal manure categories during those years, ManureDB offered a robust look into modern livestock manure nutrient concentrations, across a broader array of locations than the previously published approximations. These updates enhance benchmarking opportunities for new and existing producers and manure buyers to compare across different geographies and managements and also create an improved data source for agricultural and environmental researchers and modelers.

With the variety of management, climates, and manure storage systems, it was not surprising to have a wide range of values. Standardizing nomenclature and adding more detail collected from manure samples will improve ManureDB's future utility. Species, life stage, manure storage type, treatment, and bedding inclusion information would aid in manure characteristic comparisons. Continual additions to ManureDB will assist in the monitoring for manure nutrient trend changes and how nutrient management needs to reflect these changes. Manure production volumes are not considered currently in ManureDB and would be the other major component to determine total nutrient production on a farm.

The MWPS and ASABE both caution that while manure nutrient estimates can be helpful for designing manure storages and initial nutrient management plans, annual on‐farm samples are recommended once an animal feeding operation is operational. That recommendation still applies as these analyses reinforce the previous sentiments that manure is variable. Farms with their own annual manure analyses will be best equipped to make effective nutrient management decisions that enhance crop production as well as maintain or improve environmental quality.

## AUTHOR CONTRIBUTIONS

**Nancy L. Bohl Bormann**: Conceptualization; data curation; formal analysis; investigation; methodology; visualization; writing—original draft; writing—review and editing. **Melissa L. Wilson**: Conceptualization; data curation; funding acquisition; investigation; methodology; project administration; supervision; writing—review and editing. **Erin L. Cortus**: Conceptualization; data curation; funding acquisition; investigation; methodology; project administration; supervision; writing—review and editing. **Kevin A. T. Silverstein**: Conceptualization; methodology; project administration; software; supervision; writing—review and editing. **Kevin A. Janni**: Conceptualization; writing—review and editing. **Larry M. Gunderson**: Conceptualization; methodology; writing—review and editing. **Anna M. Cates**: Writing—review and editing. **Paulo H. Pagliari**: Writing—review and editing.

## CONFLICT OF INTEREST STATEMENT

The authors declare no conflicts of interest.

## Supporting information



Supplemental tables S1‐S5 in this manuscript display detailed US regional manure nutrient and total solids (TS) ManureDB statistics for selected animal manure categories along with MWPS and ASABE book value comparisons when available.

## Data Availability

The data for the article are available at Bohl Bormann et al. (2024a).
