# Supplementary material for: Nutrient data from US manure systems from 2012 to 2022: Spatial and temporal trends
Source: J Environ Qual. 2026 Sep 21;55(5):e70268. doi: 10.1002/jeq2.70268 (PMC13594371; doi:10.1002/jeq2.70268)
Supplement: Supplementary file 1 [file JEQ2-55-0-s001.docx]

Supplemental Material

NUTRIENT DATA FROM U.S. MANURE SYSTEMS FROM 2012-2022: SPATIAL AND TEMPORAL TRENDS

Authors: *Nancy L. Bohl Bormann^1^, Melissa L. Wilson^1^, Erin L. Cortus^2^, Kevin A.T. Silverstein^3^, Kevin A. Janni^2^, Larry M. Gunderson^4^, Anna M. Cates^1^, Paulo H. Pagliari^1^

^*^Corresponding author

^1^University of Minnesota-Twin Cities, Soil, Water, & Climate Department, 439 Borlaug Hall, 1991 Upper Buford Circle, Saint Paul, MN 55108 USA

^2^University of Minnesota-Twin Cities, Bioproducts & Biosystems Engineering Department, 210 BioAgEng Building, 1390 Eckles Ave, Saint Paul, MN 55108 USA

^3^University of Minnesota-Twin Cities, Research Computing, 599 Walter Library, 117 Pleasant St SE, Minneapolis, MN 55455

^4^Minnesota Department of Agriculture, Pesticide & Fertilizer Management, 625 Robert Street North, Saint Paul, MN 55155-2538

Number of pages: 11

Number of tables: 5

| Table S1. Descriptive statistics of as-received beef manure sample characteristics in ManureDB, for 2012 to 2022^a^. (Solid beef \| \| \| \| \| \| \| \| \| \| \| Liquid beef \| \| \| \| \| \| \| \| \| \| \|; Variable \| Median \| \| MAD^b^ \| \| RMD^c^ \| \| 25%^d^ \| 75%^e^ \| Count \| \| Variable \| Median \| \| \| MAD^b^ \| \| RMD^c^ \| \| 25%^d^ \| 75%^e^ \| \| Count; \| g kg^-1^ \| \| \| \| \| \| \| \| \| \| \| g L^-1^ \| \| \| \| \| \| \| \| \| \|; Midwest \| \| \| \| \| \| \| \| \| \| \| Midwest \| \| \| \| \| \| \| \| \| \| \|; Total N \| 7.4 \| \| 2.67 \| \| 36.1 \| \| 5.8 \| 9.5 \| 8,162 \| \| Total N \| 2.7 \| \| 3.41 \| \| \| 126 \| \| 0.576 \| 5.16 \| \| 2,928; NH_4_-N \| 1 \| \| 1.33 \| \| 133 \| \| 0.2 \| 2.3 \| 3,982 \| \| NH_4_-N \| \| 1.6 \| 2.22 \| \| \| 139 \| \| 0.1 \| 3 \| \| 1,743; P_2_O_5_ \| 4.3 \| \| 2.22 \| \| 51.7 \| \| 3 \| 6.4 \| 8,148 \| \| P_2_O_5_ \| 1.3 \| \| 1.56 \| \| \| 120 \| \| 0.4 \| 2.8 \| \| 2,928; K_2_O \| 6.3 \| \| 3.11 \| \| 49.4 \| \| 4.5 \| 9 \| 8,148 \| \| K_2_O \| 2.2 \| \| 1.63 \| \| \| 74.1 \| \| 1.1 \| 3.3 \| \| 2,928; TS \| 286 \| \| 130 \| \| 45.5 \| \| 218 \| 408 \| 8163 \| \| TS \| 43.9 \| \| 49.2 \| \| \| 112 \| \| 9.57 \| 75.4 \| \| 2,928; Northeast \| \| \| \| \| \| \| \| \| \| \| \| \| \| \| \| \| \| \| \| \| \|; Total N \| 5.74 \| \| 2.59 \| \| 45.1 \| \| 4.18 \| 7.79 \| 753 \| \| \| \| \| \| \| \| \| \| \| \| \|; NH_4_-N \| 0.275 \| \| 0.335 \| \| 122 \| \| 0.0839 \| 0.662 \| 735 \| \| \| \| \| \| \| \| \| \| \| \| \|; P_2_O_5_ \| 2.98 \| \| 1.46 \| \| 49 \| \| 2.11 \| 4.19 \| 747 \| \| \| \| \| \| \| \| \| \| \| \| \|; K_2_O \| 6.3 \| \| 4.72 \| \| 74.9 \| \| 3.58 \| 10 \| 747 \| \| \| \| \| \| \| \| \| \| \| \| \|; TS \| 256 \| \| 91.8 \| \| 35.8 \| \| 204 \| 329 \| 765 \| \| \| \| \| \| \| \| \| \| \| \| \|; Southeast \| \| \| \| \| \| \| \| \| \| \| \| \| \| \| \| \| \| \| \| \| \|; Total N \| 6.68 \| 3.26 \| \| 48.8 \| \| 4.94 \| \| 9.65 \| 757 \| \| \| \| \| \| \| \| \| \| \| \| \|; NH_4_-N \| 0.203 \| 0.198 \| \| 97.6 \| \| 0.088 \| \| 0.362 \| 475 \| \| \| \| \| \| \| \| \| \| \| \| \|; P_2_O_5_ \| 4.4 \| 2.7 \| \| 61.4 \| \| 2.86 \| \| 6.99 \| 781 \| \| \| \| \| \| \| \| \| \| \| \| \|; K2O \| 7.47 \| 5.34 \| \| 71.5 \| \| 4.33 \| \| 12.2 \| 781 \| \| \| \| \| \| \| \| \| \| \| \| \|; TS \| 354 \| 175 \| \| 49.5 \| \| 255 \| \| 518 \| 752 \| \| \| \| \| \| \| \| \| \| \| \| \|; All Regions \| \| \| \| \| \| \| \| \| \| \| All Regions \| \| \| \| \| \| \| \| \| \| \|; Total N \| 7.3 \| 2.82 \| \| 38.6 \| \| 5.59 \| \| 9.41 \| 10,023 \| \| Total N \| \| 2.4 \| 3.41 \| \| 142 \| \| 0.2 \| \| 6.2 \| 5,358 \|; NH_4_-N \| 0.7 \| 0.9 \| \| 129 \| \| 0.172 \| \| 1.9 \| 5,427 \| \| NH_4_-N \| \| 0.39 \| 0.578 \| \| 148 \| \| 0.04 \| \| 2.3 \| 3,888 \|; P_2_O_5_ \| 4.25 \| 2.3 \| \| 54.3 \| \| 2.91 \| \| 6.4 \| 10,026 \| \| P_2_O_5_ \| \| 1.3 \| 1.79 \| \| 138 \| \| 0.2 \| \| 4 \| 5,355 \|; K_2_O \| 6.49 \| 3.43 \| \| 52.9 \| \| 4.4 \| \| 9.39 \| 10,026 \| \| K_2_O \| \| 2.3 \| 2.24 \| \| 97.2 \| \| 0.9 \| \| 4.35 \| 5,348 \|; TS \| 290 \| 135 \| \| 46.6 \| \| 221 \| \| 426 \| 10,030 \| \| TS \| \| 5.2 \| 6.23 \| \| 120 \| \| 1 \| \| 56.1 \| 5,105 \|) ^a^ManureDB (Bohl Bormann et al. 2024); ^b^median absolute deviation; ^c^relative median deviation; ^d^25th percentile; ^e^75th percentile, ; ^f^Beef cows, feeder calves, and finishing cattle range of means (Lorimor et al., 2004); ^g^Beef earthen lot means (ASABE, 2005). Table S1. Descriptive statistics of as-received beef manure sample characteristics in ManureDB, for 2012 to 2022^a^, Continued. | | | | | | | | | | | | | | | | | |
| --- | --- | --- | --- | --- | --- | --- | --- | --- | --- | --- | --- | --- | --- | --- | --- | --- | --- |
| Solid beef | | | | | | |  | Liquid beef | | | | | | | | | |
| Variable | Mean | MAD^b^ | RMD^c^ | 25%^d^ | 75%^e^ | Count |  | Variable | Mean | | MAD^b^ | RMD^c^ | 25%^d^ | | 75%^e^ | | Count |
|  | g kg^-1^ | | | | |  |  |  | g L^-1^ | | | | | | |  | |
| MWPS^f^ | | | | | | |  | MWPS^f^ | | | | | | | | | |
| Total N | 3.5-05.5 | |  |  |  |  |  | Total N | 2.4-3.5 | | |  |  |  | | |  |
| NH_4_-N | 1.5-2.0 | |  |  |  |  |  | NH_4_-N | 0.8-1.0 | | |  |  |  | | |  |
| P2O5 | 2.0-3.5 | |  |  |  |  |  | P_2_O_5_ | 1.9-2.2 | | |  |  |  | | |  |
| K2O | 3.5-5.5 | |  |  |  |  |  | K_2_O | 2.9-3.1 | | |  |  |  | | |  |
| ASABE^g^ | | | | | | |  |  |  |  | |  |  |  | | |  |
| Total N | 11.8 |  |  |  |  | 1144 |  |  |  |  | |  |  |  | | |  |
| NH_4_-N | 1.0 |  |  |  |  | 1144 |  |  |  |  | |  |  |  | | |  |
| P_2_O_5_ | 11.5 |  |  |  |  | 1144 |  |  |  |  | |  |  |  | | |  |
| K_2_O | 15.1 |  |  |  |  | 1144 |  |  |  |  | |  |  |  | | |  |
| TS | 672 |  |  |  |  | 1144 |  |  |  |  | |  |  |  | | |  |
| ^a^ManureDB (Bohl Bormann et al. 2024); ^b^median absolute deviation; ^c^relative median deviation; ^d^25th percentile; ^e^75th percentile; ^f^Beef cows, feeder calves, and finishing cattle range of means (Lorimor et al., 2004); ^g^Beef earthen lot means (ASABE, 2005). | | | | | | | | | | | | | | | | | |
|  | | | | | | | | | | | | | |  | | |  |

Table S2. Descriptive statistics of as-received dairy manure sample characteristics in ManureDB, for 2012 to 2022^a^.

| Solid dairy | | | | | | |  | Liquid dairy | | | | | |  |
| --- | --- | --- | --- | --- | --- | --- | --- | --- | --- | --- | --- | --- | --- | --- |
| Variable | Median | MAD^b^ | RMD^c^ | 25%^d^ | 75%^e^ | Count |  | Variable | Median | MAD^b^ | RMD^c^ | 25%^d^ | 75%^e^ | Count |
|  | g kg^-1^ | | | | |  |  |  | g L^-1^ | | | | |  |
| Midwest | | | | | | |  | Midwest | | | | | | |
| Total N | 4.1 | 2.08 | 50.6 | 3 | 6.1 | 16,338 |  | Total N | 2.18 | 0.796 | 36.6 | 1.6 | 2.7 | 43,346 |
| NH_4_-N | 0.8 | 1.04 | 130 | 0.04 | 1.5 | 7,500 |  | NH_4_-N | 1 | 0.593 | 59.3 | 0.517 | 1.4 | 21,318 |
| P_2_O_5_ | 1.77 | 1.04 | 58.7 | 1.2 | 2.97 | 16,332 |  | P_2_O_5_ | 0.886 | 0.446 | 50.4 | 0.57 | 1.17 | 43,278 |
| K_2_O | 3.33 | 1.89 | 56.7 | 2.37 | 5.6 | 16,331 |  | K_2_O | 2.13 | 0.691 | 32.4 | 1.67 | 2.6 | 43,365 |
| TS | 263 | 155 | 58.9 | 171 | 391 | 16,921 |  | TS | 41.6 | 24.5 | 58.8 | 25.9 | 58.9 | 43,691 |
| Northeast | | | | | | |  | Northeast | | | | | | |
| Total N | 4.23 | 1.39 | 32.9 | 3.41 | 5.35 | 5,925 |  | Total N | 2.32 | 1.04 | 44.9 | 1.5 | 2.94 | 4,599 |
| NH_4_-N | 0.876 | 0.695 | 79.4 | 0.426 | 1.37 | 5,817 |  | NH_4_-N | 1.04 | 0.523 | 50.4 | 0.636 | 1.36 | 4,532 |
| P_2_O_5_ | 1.88 | 0.777 | 41.3 | 1.44 | 2.61 | 6,022 |  | P_2_O_5_ | 0.909 | 0.446 | 49.1 | 0.534 | 1.17 | 4,664 |
| K_2_O | 4.05 | 2.12 | 52.4 | 2.82 | 5.88 | 5,937 |  | K_2_O | 2.18 | 0.955 | 43.8 | 1.53 | 2.82 | 4,664 |
| TS | 212 | 105 | 49.6 | 150 | 306 | 6,047 |  | TS | 47.3 | 34.3 | 72.4 | 24.4 | 70.5 | 4,721 |
| Southeast | | | | | | |  | Northern Plains | | | | | | |
| Total N | 5 | 3.13 | 62.7 | 3.25 | 8.09 | 2,621 |  | Total N | 2.2 | 0.689 | 31.4 | 1.75 | 2.66 | 626 |
| NH_4_-N | 0.184 | 0.196 | 106 | 0.0719 | 0.364 | 1,513 |  | NH_4_-N | 1.07 | 0.489 | 45.7 | 0.785 | 1.4 | 361 |
| P_2_O_5_ | 2.78 | 2.22 | 79.9 | 1.58 | 4.87 | 3,017 |  | P_2_O_5_ | 0.724 | 0.297 | 41 | 0.503 | 0.91 | 627 |
| K_2_O | 4.09 | 4.15 | 101 | 1.81 | 7.95 | 3,017 |  | K_2_O | 2.01 | 0.539 | 26.8 | 1.6 | 2.31 | 627 |
| TS | 297 | 185 | 62.2 | 183 | 442 | 2,636 |  | TS | 40.5 | 22.2 | 54.8 | 24.5 | 54.9 | 625 |

^a^ManureDB (Bohl Bormann et al. 2024); ^b^median absolute deviation; ^c^relative median deviation; ^d^25th percentile; ^e^75th percentile; ^f^Dairy cow, heifer, calf, and herd range of means (Lorimor et al., 2004); ^g^Dairy scraped concrete, earthen lots means (ASABE, 2005); ^h^Dairy lagoon effluent, slurry (ASABE, 2005).

| Table S2. Descriptive statistics of as-received dairy manure sample characteristics in ManureDB, for 2012 to 2022^a^, Continued. Solid dairy \| \| Liquid dairy | | | | | | | | | | | | | | | | | | |
| --- | --- | --- | --- | --- | --- | --- | --- | --- | --- | --- | --- | --- | --- | --- | --- | --- | --- | --- |
| Variable | Median | MAD^b^ | RMD^c^ | 25%^d^ | 75%^e^ | Count |  | Variable | | Median | | MAD^b^ | RMD^c^ | 25%^d^ | | 75%^e^ | Count | |
|  | g kg^-1^ | | | | |  |  | | | g L^-1^ | | | | | | | |  |
| Southwest | | | | | | |  | Southeast | | | | | | | | | | |
| Total N | 12 | 7.86 | 65.8 | 6.6 | 17.2 | 870 |  | Total N | | 0.47 | 0.635 | | 135 | 0.134 | 1.37 | | | 5,794 |
| NH_4_-N | 0.192 | 0.0245 | 12.7 | 0.176 | 0.209 | 1435 |  | NH_4_-N | | 0.0742 | 0.0971 | | 131 | 0.019 | 0.182 | | | 2,816 |
| P_2_O_5_ | 7.9 | 6.23 | 78.8 | 3.5 | 11.7 | 872 |  | P_2_O_5_ | | 0.379 | 0.435 | | 115 | 0.128 | 0.992 | | | 7,080 |
| K_2_O | 16.5 | 17.2 | 104 | 4.2 | 26.7 | 872 |  | K_2_O | | 1.11 | 0.993 | | 89.7 | 0.539 | 2.03 | | | 7,079 |
| TS | 678 | 309 | 45.5 | 363 | 838 | 871 |  | TS | | 35.5 | 38.4 | | 108 | 8.63 | 59.6 | | | 1,805 |
|  | | | | | | |  | Southwest | | | | | | | | | | |
|  |  |  |  |  |  |  |  | Total N | | 0.476 | 0.345 | | 72.6 | 0.266 | 0.758 | | | 1,485 |
|  |  |  |  |  |  |  |  | NH_4_-N | | 0.292 | 0.197 | | 67.5 | 0.155 | 0.418 | | | 1,435 |
|  |  |  |  |  |  |  |  | P_2_O_5_ | | 0.173 | 0.108 | | 62.6 | 0.113 | 0.298 | | | 1,488 |
|  |  |  |  |  |  |  |  | K_2_O | | 0.803 | 0.484 | | 60.3 | 0.466 | 1.12 | | | 1,489 |
|  |  |  |  |  |  |  |  | TS | | 8.1 | 8.35 | | 103 | 3.38 | 15.8 | | | 120 |
| All Regions | | | | | | |  | All Regions | | | | | | | | | | |
| Total N | 4.26 | 2.03 | 47.7 | 3.11 | 6.24 | 26,099 |  | Total N | | 2.05 | 0.964 | | 47.1 | 1.3 | 2.62 | | | 56,541 |
| NH_4_-N | 0.725 | 0.853 | 118 | 0.193 | 1.38 | 15,006 |  | NH_4_-N | | 0.9 | 0.741 | | 82.4 | 0.246 | 1.3 | | | 31,146 |
| P_2_O_5_ | 1.9 | 1.15 | 60.3 | 1.3 | 3.2 | 26,590 |  | P_2_O_5_ | | 0.804 | 0.528 | | 65.7 | 0.426 | 1.14 | | | 57,833 |
| K_2_O | 3.62 | 2.26 | 62.3 | 2.43 | 6.1 | 26,504 |  | K_2_O | | 2.06 | 0.826 | | 40.2 | 1.45 | 2.6 | | | 57,907 |
| TS | 258 | 152 | 59.1 | 167 | 388 | 26,822 |  | TS | | 41.4 | 26.1 | | 63 | 24.3 | 59.4 | | | 51,655 |
| ^a^ManureDB (Bohl Bormann et al. 2024); ^b^median absolute deviation; ^c^relative median deviation; ^d^25th percentile; ^e^75th percentile; ^f^Dairy cow, heifer, calf, and herd range of means (Lorimor et al., 2004); ^g^Dairy scraped concrete, earthen lots means (ASABE, 2005); ^h^Dairy lagoon effluent, slurry (ASABE, 2005). | | | | | | | | | | | | | | | | | | |
| Table S2. Descriptive statistics of as-received dairy manure sample characteristics in ManureDB, for 2012 to 2022^a^, Continued. | | | | | | | | | | | | | | | | | | |
| Solid dairy | | | | | | |  | | Liquid dairy | | | | | | | | |  |
| Variable | Mean | MAD^b^ | RMD^c^ | 25%^d^ | 75%^e^ | Count |  | | Variable | Mean | MAD^b^ | | RMD^c^ | 25%^d^ | 75%^e^ | | | Count |
|  | g kg^-1^ | | | | |  |  | |  | g L^-1^ | | | | | | | |  |
| MWPS^f^ | | | | | | |  | | MWPS^f^ | | | | | | | | | |
| Total N | 4.5-5.0 | |  |  |  |  |  | | Total N | 3.2-3.8 | | |  |  |  | | |  |
| NH_4_-N | 1.0 |  |  |  |  |  |  | | NH_4_-N | 0.6-0.7 | | |  |  |  | | |  |
| P_2_O_5_ | 1.5-2.0 | |  |  |  |  |  | | P_2_O_5_ | 1.7-1.8 | | |  |  |  | | |  |
| K_2_O | 2.5-3.5 | |  |  |  |  |  | | K_2_O | 2.3-3.4 | | |  |  |  | | |  |
| ASABE^g^ | | | | | | |  | | ASABE^h^ | | | | | | | | | |
| Total N | 5.3-7.0 | |  |  |  | 666 |  | | Total N | 0.7, 3.0 | | |  |  |  | | | 2,707 |
| NH_4_-N |  |  |  |  |  | 666 |  | | NH_4_-N | 0.8, 1.4 | | |  |  |  | | | 2,707 |
| P_2_O_5_ | 3.0-5.7 | |  |  |  | 666 |  | | P_2_O_5_ | 0.4, 3.0 | | |  |  |  | | | 2,707 |
| K_2_O | 4.8-8.0 | |  |  |  | 666 |  | | K_2_O | 1.3, 4.8 | | |  |  |  | | | 2,707 |
| TS | 250, 460 | |  |  |  | 666 |  | | TS | 20, 80 | | |  |  |  | | | 2,707 |
| ^a^ManureDB (Bohl Bormann et al. 2024); ^b^median absolute deviation; ^c^relative median deviation; ^d^25th percentile; ^e^75th percentile; ^f^Dairy cow, | | | | | | | | | | | | | | | | | | |
| heifer, calf, and herd range of means (Lorimor et al., 2004); ^g^Dairy scraped concrete, earthen lots means (ASABE, 2005); ^h^Dairy lagoon effluent, slurry means (ASABE, 2005). | | | | | | | | | | | | | | | | | | |

Table S3. Descriptive statistics of as-received broiler and layer chicken manure sample characteristics in ManureDB, for 2012 to 2022_a_.

| Solid broiler | | | | | | |  | Solid layer | | | | | | |
| --- | --- | --- | --- | --- | --- | --- | --- | --- | --- | --- | --- | --- | --- | --- |
| Variable | Median | MAD^b^ | RMD^c^ | 25%^d^ | 75%^e^ | Count |  | Variable | Median | MAD^b^ | RMD^c^ | 25%^d^ | 75%^e^ | Count |
|  | g kg^-1^ | | | | |  |  |  | g kg^-1^ | | | | |  |
| Northeast | | | | | | |  | Northeast | | | | | | |
| Total N | 27.8 | 6.39 | 22.9 | 23.7 | 32.2 | 630 |  | Total N | 22.8 | 9.89 | 43.4 | 17 | 30.5 | 507 |
| NH_4_-N | 3.43 | 2.07 | 60.5 | 2.23 | 5.06 | 626 |  | NH_4_-N | 4.19 | 2.41 | 57.7 | 2.65 | 5.89 | 507 |
| P_2_O_5_ | 20.5 | 7.71 | 37.5 | 16 | 26.9 | 630 |  | P_2_O_5_ | 22.9 | 11.3 | 49.5 | 16.4 | 31.6 | 507 |
| K_2_O | 24 | 7.6 | 31.6 | 18.9 | 29.1 | 630 |  | K_2_O | 15.7 | 7.83 | 49.9 | 10.8 | 21.3 | 507 |
| TS | 711 | 103 | 14.5 | 639 | 779 | 630 |  | TS | 541 | 224 | 41.4 | 372 | 684 | 507 |
| Southeast | | | | | | |  | Southeast | | | | | | |
| Total N | 28.7 | 5.63 | 19.6 | 24.5 | 32.2 | 17,846 |  | Total N | 21.3 | 9.22 | 43.4 | 14.8 | 27.3 | 2,797 |
| NH_4_-N | 4.11 | 1.63 | 39.6 | 3.04 | 5.23 | 6,238 |  | NH_4_-N | 2.18 | 1.3 | 59.7 | 1.4 | 3.13 | 1,349 |
| P_2_O_5_ | 25.1 | 6.99 | 27.9 | 20.6 | 30 | 17,851 |  | P_2_O_5_ | 27.8 | 10.9 | 39.4 | 21 | 35.8 | 2,862 |
| K_2_O | 28.4 | 6.4 | 22.6 | 24.1 | 32.7 | 17,851 |  | K_2_O | 20.8 | 7.41 | 35.6 | 15.8 | 25.7 | 2,862 |
| TS | 748 | 70.6 | 9.43 | 692 | 789 | 9487 |  | TS | 698 | 159 | 22.9 | 552 | 783 | 1732 |
| All Regions | | | | | | |  | All Regions | | | | | | |
| Total N | 28.7 | 5.66 | 19.7 | 24.5 | 32.2 | 18,604 |  | Total N | 21.6 | 9.26 | 42.9 | 15.6 | 28 | 3,526 |
| NH_4_-N | 4.08 | 1.68 | 41.2 | 2.97 | 5.24 | 6,973 |  | NH_4_-N | 2.61 | 1.7 | 65 | 1.6 | 4.02 | 1,965 |
| P_2_O_5_ | 25 | 7.06 | 28.3 | 20.4 | 29.9 | 18,609 |  | P_2_O_5_ | 26.9 | 11.3 | 42.1 | 20 | 35.3 | 3,591 |
| K_2_O | 28.3 | 6.5 | 23 | 23.9 | 32.6 | 18,609 |  | K_2_O | 20.1 | 7.96 | 39.6 | 14.4 | 25.2 | 3,591 |
| TS | 746 | 72.9 | 9.77 | 688 | 789 | 10,245 |  | TS | 664 | 184 | 27.7 | 500 | 770 | 2,461 |
| ^a^ManureDB (Bohl Bormann et al. 2024); ^b^median absolute deviation; ^c^relative median deviation; ^d^25th percentile; ^e^75th percentile; | | | | | | | | | | | | | | |
| ^f^Broiler means (Lorimor et al., 2004); ^g^Poultry broiler litter means (ASABE, 2005); ^h^ Layer means (Lorimor et al., 2004); ^i^Leghorn hen means (ASABE, 2005). | | | | | | | | | | | | | | |
| Table S3. Descriptive statistics of as-received broiler and layer chicken manure sample characteristics in ManureDB, for 2012 to 2022^a^, Continued. | | | | | | | | | | | | | | |
| Solid broiler | | | | | | |  | Solid layer | | | | | | |
| Variable | Mean | MAD^b^ | RMD^c^ | 25%^d^ | 75%^e^ | Count |  | Variable | Mean | MAD^b^ | RMD^c^ | 25%^d^ | 75%^e^ | Count |
|  | g kg^-1^ | | | | |  |  |  | g kg^-1^ | | | | |  |
| MWPS^f^ | | | | | | |  | MWPS^h^ | | | | | | |
| Total N | 23 |  |  |  |  |  |  | Total N | 17 |  |  |  |  |  |
| NH_4_-N | 6 |  |  |  |  |  |  | NH_4_-N | 6 |  |  |  |  |  |
| P_2_O_5_ | 26.5 |  |  |  |  |  |  | P_2_O_5_ | 25.5 |  |  |  |  |  |
| K_2_O | 18 |  |  |  |  |  |  | K_2_O | 13 |  |  |  |  |  |
| ASABE^g^ | | | | | | |  | ASABE^i^ | | | | | | |
| Total N | 37.3 |  |  |  |  | 95 |  | Total N | 18.5 |  |  |  |  | 48 |
| NH_4_-N | 7.5 |  |  |  |  | 95 |  | NH_4_-N | 8.8 |  |  |  |  | 48 |
| P_2_O_5_ | 13.7 |  |  |  |  | 95 |  | P_2_O_5_ | 27.7 |  |  |  |  | 48 |
| K_2_O | 16.5 |  |  |  |  | 95 |  | K_2_O | 15.7 |  |  |  |  | 48 |
| TS | 700 |  |  |  |  | 95 |  | TS | 400 |  |  |  |  | 48 |
|  |  |  |  |  |  |  |  |  |  |  |  |  |  |  |
| ^a^ManureDB (Bohl Bormann et al. 2024); ^b^median absolute deviation; ^c^relative median deviation; ^d^25th percentile; | | | | | | | | | | | | | | |
| ^e^75th percentile; ^f^Broiler means (Lorimor et al., 2004); ^g^Poultry broiler litter means (ASABE, 2005); ^h^ Layer means (Lorimor et al., 2004); | | | | | | | | | | | | | | |
| ^i^Leghorn hen means (ASABE, 2005). | | | | | | | | | | | | | | |
|  | | | | | | | | | | | | | | |

Table S4. Descriptive statistics of as-received solid turkey manure sample characteristics in ManureDB, for 2012 to 2022^a^.

| Variable | Median | MAD^b^ | RMD^c^ | 25%^d^ | 75%^e^ | Count |
| --- | --- | --- | --- | --- | --- | --- |
|  | g kg^-1^ | | | | |  |
| Midwest | | | | | | |
| Total N | 25.6 | 7.13 | 27.8 | 20.8 | 30.4 | 1,713 |
| NH_4_-N | 7.1 | 2.97 | 41.8 | 5.1 | 9.1 | 1,007 |
| P_2_O_5_ | 23.2 | 9.93 | 42.8 | 17 | 30.8 | 1,713 |
| K_2_O | 19.7 | 6.08 | 30.9 | 15.7 | 23.9 | 1,713 |
| TS | 627 | 117 | 18.7 | 544 | 703 | 1,713 |
| Southeast | | | | | | |
| Total N | 24.6 | 7.72 | 31.4 | 19.3 | 29.8 | 3,659 |
| NH_4_-N | 5.01 | 1.72 | 34.3 | 3.78 | 6.11 | 2,261 |
| P_2_O_5_ | 26.1 | 7.78 | 29.8 | 20.6 | 31.1 | 3,662 |
| K_2_O | 21 | 6.4 | 30.4 | 15.9 | 24.9 | 3,662 |
| TS | 702 | 100 | 14.3 | 627 | 764 | 3,668 |
| All Regions | | | | | | |
| Total N | 25 | 7.48 | 29.9 | 19.9 | 30 | 5,631 |
| NH_4_-N | 5.4 | 2.25 | 41.7 | 3.94 | 6.97 | 3,526 |
| P_2_O_5_ | 25.3 | 8.82 | 34.8 | 19.1 | 31 | 5,634 |
| K_2_O | 20.6 | 6.52 | 31.7 | 15.8 | 24.7 | 5,634 |
| TS | 677 | 115 | 17 | 593 | 748 | 5,640 |
| MWPS^f^ | Mean |  | | | | |
| Total N | 20 |  |  |  |  |  |
| NH_4_-N | 4.0 |  |  |  |  |  |
| P_2_O_5_ | 25 |  |  |  |  |  |
| K_2_O | 15 |  |  |  |  |  |
| ASABE^g^ | Mean | | | | |  |
| Total N | 21.8 |  |  |  |  |  |
| NH_4_-N | - |  |  |  |  |  |
| P_2_O_5_ | 7.6 |  |  |  |  |  |
| K_2_O | 14.8 |  |  |  |  |  |
| TS | - |  |  |  |  |  |

^a^ManureDB (Bohl Bormann et al. 2024); ^b^median absolute deviation; ^c^relative median deviation; ^d^25th percentile; ^e^75th percentile; ^f^Tom, hen turkey means (Lorimor et al., 2004); ^g^Turkey litter means (ASABE, 2005).

Table S5. Descriptive statistics of as-received liquid swine manure sample characteristics in ManureDB, for 2012 to 2022^a^.

| Variable | Median | MAD^b^ | RMD^c^ | 25%^d^ | 75%^e^ | Count |
| --- | --- | --- | --- | --- | --- | --- |
|  | g L^-1^ | | | | |  |
| Midwest | | | | | | |
| Total N | 4.9 | 2.52 | 51.4 | 2.99 | 6.5 | 78,150 |
| NH_4_-N | 3 | 1.66 | 55.4 | 1.8 | 4.1 | 34,156 |
| P_2_O_5_ | 2.2 | 1.48 | 67.4 | 1.2 | 3.2 | 78,148 |
| K_2_O | 3.3 | 1.78 | 53.9 | 2 | 4.3 | 78,151 |
| TS | 39.3 | 30.2 | 77 | 19.4 | 60.4 | 78,151 |
| Northeast | | | | | | |
| Total N | 3.25 | 1.99 | 61.1 | 2.06 | 4.8 | 633 |
| NH_4_-N | 2.09 | 1.18 | 56.3 | 1.34 | 2.95 | 624 |
| P_2_O_5_ | 1.17 | 1.41 | 120 | 0.403 | 2.93 | 637 |
| K_2_O | 2.13 | 1.32 | 61.8 | 1.38 | 3.41 | 637 |
| TS | 19.7 | 19.7 | 100 | 8.82 | 44.1 | 638 |
| Northern Plains | | | | | | |
| Total N | 4.6 | 2.97 | 64.5 | 2.4 | 6.47 | 754 |
| NH_4_-N | 3.18 | 2.4 | 75.6 | 1.48 | 4.69 | 728 |
| P_2_O_5_ | 2.6 | 2.12 | 81.5 | 0.71 | 3.8 | 754 |
| K_2_O | 2.74 | 1.73 | 63.3 | 1.5 | 3.8 | 751 |
| TS | 39 | 35.6 | 91.2 | 15.4 | 63 | 754 |
| Southeast | | | | | | |
| Total N | 0.32 | 0.183 | 56.4 | 0.21 | 0.46 | 12,0108 |
| NH_4_-N | 0.003 | 0.00468 | 142 | 0.000633 | 0.0311 | 2,968 |
| P_2_O_5_ | 0.09 | 0.0401 | 45.7 | 0.0637 | 0.12 | 120,420 |
| K_2_O | 0.67 | 0.35 | 52.6 | 0.446 | 0.925 | 120,420 |
| TS | 4.53 | 3.66 | 80.9 | 2.38 | 8.14 | 2,424 |
| All Regions | | | | | | |
| Total N | 0.52 | 0.54 | 104 | 0.29 | 3.9 | 199,767 |
| NH_4_-N | 2.8 | 1.84 | 65.7 | 1.5 | 4 | 38,579 |
| P_2_O_5_ | 0.13 | 0.12 | 89.6 | 0.079 | 1.79 | 200,079 |
| K_2_O | 1 | 0.84 | 83.7 | 0.585 | 2.66 | 200,069 |
| TS | 38.1 | 31.1 | 81.7 | 17.6 | 59.7 | 82,089 |

^a^ManureDB (Bohl Bormann et al. 2024); ^b^median absolute deviation; ^c^relative median deviation; ^d^25th percentile; ^e^75th percentile; ^f^Swine farrowing, nursery, grow-finish deep pit, grow-finish wet/dry feeder, grow-finish earthen pit, breeding-gestation, farrow-finish, farrow-feeder means (Lorimor et al., 2004); ^g^Swine finisher-slurry wet-dry feeders, slurry dry feeders, flush building, agitated solids and water, lagoon surface water, lagoon sludge means (ASABE, 2005).

| Table S5. Descriptive statistics of as-received liquid swine manure sample characteristics in ManureDB, for 2012 to 2022^a^, Continued. | | | | | | | |
| --- | --- | --- | --- | --- | --- | --- | --- |
| Variable | Mean | MAD^b^ | | RMD^c^ | 25%^d^ | 75%^e^ | Count |
|  | g L^-1^ | | | | | |  |
| MWPS^f^ | | | | | | | |
| Total N | 1.8-7 | |  | |  |  |  |
| NH_4_-N | 1-4.7 | |  | |  |  |  |
| P_2_O_5_ | 1.4-5.3 | |  | |  |  |  |
| K_2_O | 1.3-4.8 | |  | |  |  |  |
| ASABE^g^ | | | | | | |  |
| Total N | 0.6-7.0 | |  | |  |  | 481+ |
| NH_4_-N | 0.4-5 | |  | |  |  | 481+ |
| P_2_O_5_ | 0.5-5.7 | |  | |  |  | 481+ |
| K_2_O | 0.7-2.9 | |  | |  |  | 481+ |
| TS | 4-100 | |  | |  |  | 481+ |

^a^ManureDB (Bohl Bormann et al. 2024); ^b^median absolute deviation; ^c^relative median deviation; ^d^25th percentile; ^e^75th percentile; ^f^Swine farrowing, nursery, grow-finish deep pit, grow-finish wet/dry feeder, grow-finish earthen pit, breeding-gestation, farrow-finish, farrow-feeder means (Lorimor et al., 2004); ^g^Swine finisher-slurry wet-dry feeders, slurry dry feeders, flush building, agitated solids and water, lagoon surface water, lagoon sludge means (ASABE, 2005).
